# Author Correction: Modelling and predicting the spatio‑temporal spread of COVID‑19, associated deaths and impact of key risk factors in England

**DOI:** 10.1038/s41598-021-97282-8

**Published:** 2021-08-31

**Authors:** B. Sartorius, A. B. Lawson, R. L. Pullan

**Affiliations:** 1 Department of Disease Control, Faculty of Tropical and Infectious Diseases, London School of Hygiene and Tropical Medicine, Keppel Street, Bloomsbury, London, WC1E 7HT UK; 2 Centre for Tropical Medicine and Global Health, Nuffield Department of Medicine, University of Oxford, Oxford, UK; 3 Department of Health Metrics Sciences, School of Medicine, University of Washington, Seattle, WA USA; 4 Department of Public Health Sciences, Medical University of South Carolina, Charleston, SC 29425 USA

Correction to: *Scientific Reports* https://doi.org/10.1038/s41598-021-83780-2, Published online 08 March 2021

The original version of this Article contained an error in the Introduction where,

“We utilise weekly MSOA level population mobility data against observed confirmed COVID-19 case data to assess the impact mobility reduction at small-area scale has had on case transmission, and counterfactually what the magnitude may have been under the scenario of no mobility loss.”

now reads:

“We utilise weekly Clinical Commission Group (CCG) level population mobility data against observed confirmed COVID-19 case data to assess the impact mobility reduction at small-area scale has had on case transmission, and counterfactually what the magnitude may have been under the scenario of no mobility loss.”

In addition, in Table 2, the Spatial resolution was incorrectly given in the column “Model component”, rows “Covariate (daily varying)” and “Covariates (annual estimates, weekly fixed), Elderly population proportion living in deprivation”. The correct and incorrect values appear below.

Incorrect:

**Table:** 

Model component	Variable	Source (open access unless indicated*)	Spatial resolution	Temporal resolution	Compartment
Covariate (daily varying)	Mobility (population movement index)	Oxford COVID-19 impact monitor (Cuebiq) (https://www.oxford-covid-19.com/)	MSOA	Daily	Cases
Covariates (annual estimates, weekly fixed)	Elderly population proportion living in deprivation	Public Health England – Local Health: (https://www.localhealth.org.uk/)	MPSA	2019	Deaths

Correct:

**Table:** 

Model component	Variable	Source (open access unless indicated*)	Spatial resolution	Temporal resolution	Compartment
Covariate (daily varying)	Mobility (population movement index)	Oxford COVID-19 impact monitor (Cuebiq) (https://www.oxford-covid-19.com/)	Clinical Commission Group (CCG)	Daily	Cases
Covariates (annual estimates, weekly fixed)	Elderly population proportion living in deprivation	Public Health England – Local Health: https://www.localhealth.org.uk	MSOA	2019	Deaths

Finally, in the Methods section, under the subheadings ‘Data’, and ‘Data analysis”,

“Daily population movement data by MSOA was extracted from the COVID-19 Impact monitor (https://www.oxford-covid-19.com/).”

now reads:

“Daily population movement data by Clinical Commission Group (CCG) was extracted from the COVID-19 Impact monitor (https://www.oxford-covid-19.com/). Weekly mobility in a given MSOA where assumed to be the same as the weekly mobility in the higher level CCG containing most of that MSOA.”

“Here *b* = (*b*_0_, *b*_1_, *b*_2_) is the vector of regression coefficients for the intercept (representing the log-transformed baseline transmission rate across all locations), mobility represents the observed weekly mobility by MSOA;”

now reads:

“Here *b* = (*b*_0_, *b*_1_, *b*_2_) is the vector of regression coefficients for the intercept (representing the log-transformed baseline transmission rate across all locations), mobility represents the observed weekly mobility in a given MSOA based on the mobility in the CCG containing most of that MSOA;”

The original Article has been corrected.

